# Repeated Load Triaxial Testing of Recycled Excavation Materials Blended with Recycled Phyllite Materials

**DOI:** 10.3390/ma15020621

**Published:** 2022-01-14

**Authors:** Solomon Adomako, Christian John Engelsen, Rein Terje Thorstensen, Diego Maria Barbieri

**Affiliations:** 1 Department of Engineering and Science, University of Agder, 4879 Grimstad, Norway; rein.t.thorstensen@uia.no; 2 Department of Building and Infrastructure, SINTEF Community, 0314 Oslo, Norway; christianjohn.engelsen@sintef.no; 3 Department of Civil and Environmental Engineering, Norwegian University of Science and Technology (NTNU), 7491 Trondheim, Norway; diego.barbieri@ntnu.no

**Keywords:** recycled excavation materials, recycled phyllite materials, resilient modulus, permanent deformation

## Abstract

Recycled Excavation Materials (REM) are becoming viable alternative construction resources due to their economic benefits. However, REM may be composed of weak rocks, e.g., phyllites, limiting the use in a base layer. The present paper attempts to further the knowledge of the mechanical performance of REM by performing Repeated Load Triaxial Tests (RLTT). REM are mixed with Recycled Phyllite Materials (RPM) in systematic blends of 0%, 25%, 50%, and 100%. The batches’ resilient modulus (*M_R_*) and permanent deformation (PD) characteristics were assessed to establish the maximum RPM allowed into REM while maintaining the required performance. Hicks and Monismith’s and Uzan’s models were used to characterize the stiffness behavior. A wide variation in the stiffness between the two materials was observed. Batches comprised of 0% RPM–100% REM and 25% RPM–75% REM showed high stiffness performance. The Coulomb model assessed the PD behavior, and the results showed a similar response for all batches. Unlike the stiffness, blended mixtures did not show sensitivity to increased RPM content in the PD. This study may help end-users to understand the performance of REM given the documented threshold on the allowable quantity of RPM in REM.

## 1. Introduction

Construction and demolition waste (CDW) is by far the heaviest and voluminous waste stream, accounting for 35% of total waste produced globally [1]. However, thanks to environmental protection legislations and recycling technology, the operations of recovering, and reusing recycled aggregates (RA) from CDW has been successful. As a result, the application of RA in civil construction and performance has been studied for many years [2,3,4,5]. Given the origin and composition of CDW, RA mainly recovered and used in construction are recycled masonry aggregates (RMA), recycled concrete aggregates (RCA), mixed recycled aggregates (MRA), reclaimed asphalt pavement (RAP), and construction and demolition recycled aggregates (CDRA) [6]. RMA is sourced from crushed rubble, RCA is obtained from demolished concrete structures, whereas MRA consists of mixtures of RMA, ceramic tile, bricks and RCA. RAP is asphalt-based, and CDRA primarily consists of plastic, glass, wood, etc. [6].

Within the framework of CDW, little attention is given to excavation materials (soil and rock). In Europe, for example, the challenge of implementing a proper traceable system has made it difficult to determine the precise volume generated every year. In addition, today’s viable technology for recycling construction waste materials is more focused on waste components such as glass, concrete, bricks, and wood [1,7]. However, the success of applying recycled excavation materials is demonstrated in big projects such as the Gotthard base tunnel in Switzerland [8].

In Western Norway, Velde’s modern aggregates recycling plant produces recycled aggregates (both soil and rocks) from excavation materials (REM), and occasionally phyllite materials are encountered [9]. Phyllites are low-grade metamorphic rocks characterized by phyllosilicates (mica and chlorite), and they usually have low engineering properties [10,11]. In addition, they are anisotropic, and weak planes develop following a preferred orientation, making it easier for a fracture to occur along the direction of the planar orientation [12]. Nevertheless, phyllites with good geometrical and mechanical properties may be applied in cement concrete [13].

Given the overall properties of RA, REM produced at Velde meets a range of technical requirements and is environmentally and economically efficient. In particular, good Los Angeles (LA) and micro-Deval (MD) performance are reported [9]. A comprehensive study of mixing different levels of recycled phyllite materials (RPM) with REM to assess the mechanical properties (LA and MD) and identify mechanically weak minerals was performed [14]. The results showed that intermixing RPM significantly affected the MD at high mixing levels (˃40%). In addition, a good correlation was found between increased peaks of phyllosilicate minerals (mica and chlorites) when increasing RPM content. Hence the acceptable amount of RPM into REM was found. Generally, geological properties, particularly mineralogy, are reported to affect rock’s LA and MD performance significantly [11]. In other studies, 50% of REM was incorporated into RCA obtained from CDW for concrete production [15]. The study showed that LA and MD performance of the mix were 30% and 20%, respectively. In addition, compressive strength after 28 days of three samples produced from the mix was reported in the region 57–65 MPa, and the acid-solubility was within 4–7%, which was reasonably lower than RCA produced from a single source of concrete rubble. REM use did not influence the physical characteristics, i.e., slump and density of concrete products [15]. Detailed technical assessment of REM in India showed good technical properties, i.e., specific gravity, LA, bulk density, soundness, and therefore these materials are suitable for a wide range of construction activities [16].

Despite this notable performance of REM, their potential application in unbound construction is not fully used in Norway due to limited research to characterize the behavior under repeated traffic. For instance, this type of mechanical response can be characterized by means of Repeated Load Triaxial Test (RLTT). Given this, REM may be applied as unbound granular material (UGM) in road pavement’s base and subbase layers.

The RLTT investigates the mechanical performance of UGM in terms of resilient modulus (*M_R_*) and resistance to permanent deformation (PD) [17,18,19,20]. In addition, the test simulates the behavior of UGM under repeated traffic loads in real applications [21]. UGM generally show anisotropic and nonlinear characteristics [22,23]. Therefore, the *M_R_* parameter adequately describes the behavior of these materials under cyclic compressive loads. In UGM, deformation behavior is classified by elastic (recoverable) and permanent (irrecoverable). Regarding the first behavior, high *M_R_* means high stiffness and is usually related to high load bearing capacity. Furthermore, an increase in confining stress usually leads to an increase in *M_R_* [24]. On the other hand, an excessive PD produces issues such as rutting, which is commonly related to high levels of deviatoric stress compared with the levels of triaxial stress. 

The *M_R_* and PD behavior generally depends on several factors, including gradation, moisture content, fines content, grain size and shape, stress history, loading frequency, and stress level [24,25,26,27]. Given these factors, stress values and moisture content represent the most sensitive conditions with a significant effect on the elastic and plastic deformation properties.

The behavior of RA based on mixing levels and acceptable thresholds for sufficient stiffness and PD performance by RLTT is reported in several studies. For example, 25% of crushed brick (CB) blended with RCA and crushed rock (CR) showed optimal stiffness and PD characteristics, and therefore, the mixture makes a good alternative for subbase material [28]. A similar study reported that 15% RAP and 85% RCA showed sufficient stiffness and deformation response at optimum moisture contents of 59–78%, compared with mixtures of increased RAP content at 30%, 50%, and 100% [29]. In conclusion, the authors emphasized the importance of respecting the mixing threshold of RAP to achieve an acceptable performance. The stiffness and PD behavior of RCA substituted with recycled clay masonry at 10–30% content was studied, and the result showed that the stiffness decreased while permanent strain increased with increased range of clay masonry, and the mixtures showed sensitivity to moisture content [30].

Regarding mixtures comprising of CDRA content, a study on the stiffness and PD behavior of mixtures of RCA and CR mixed with constant 1% crumb rubber and 1–5% of crushed glass showed that the mixtures were sensitive to increased content of crushed glass as both stiffness and PD improved [31]. In view of this, the authors recommended that approximately 1% crumb rubber and 5% crushed glass should be the optimum mixing level for both RCA and CR for base and subbase applications. In a similar study, different mix compositions of 10–50% recycled glass (5 mm) and CR were produced for stiffness and PD investigations [32]. The results showed that up to 30% recycled glass is regarded as optimum to be added to CR, considering that stiffness and PD behavior showed sufficient response just like natural aggregates. In addition, the authors mentioned that the degree of breakdown of the blends was within the acceptable limit as required of pavement subbase material. In another study, a recycled glass of 0–5 mm was mixed by volume with limestone 0–20 mm for stiffness and PD evaluation [33]. Different mixtures were prepared, but the optimum mix for sufficient stiffness and PD behavior was 25% recycled glass and 75% limestone. The authors observed that low traffic stress state corresponding to bulk stress of 82 and 276 kPa had a minor impact and all mixtures studied in these stress state performed similarly. However, a decreasing stiffness trend was observed at high-stress levels [33]. A study that evaluated the frictional strength properties of crushed glass, kaolin and fine quarry blends showed that frictional properties of the soil increased with increased crushed glass content, making the mix suitable for backfilling and embarkment applications [34]. Mixtures composed of glass gullet and caliche-weathered limestone showed that increasing the amount of glass gullet makes the combinations suitable for subbase applications [35].

Given the reported studies and previous research on RA, the almost non-existent research and knowledge of the stiffness and deformation behavior of REM and RPM mixes under repeated loads limit the use of REM composed of occasional amount of weak rocks. Unfortunately, this makes REM complete waste materials suffering the fate of landfill, which is not suitable from an environmental point of view. In addition, skepticism by end-users about the potentials of REM increases. Given that the stiffness and deformation behavior of blended mixtures composed of recycled materials vary, it is essential to assess the materials under consideration in the present study. Hence, the objective of the present study is to evaluate the performance of REM and RPM mixes in RLTT tests to establish an acceptable amount of phyllite or the content of weak rocks which may be present in REM. Finally, the findings may promote the use of REM as road construction materials.

## 2. Materials and Methods

**Figure 1:**
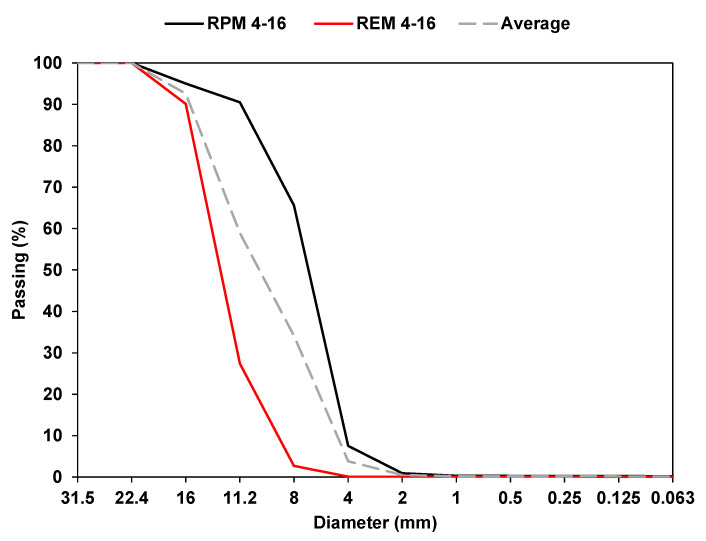
Grain size distribution curves for REM and RPM.

The materials used in the investigation were produced by Velde AS located in Sandnes (Norway). Both REM and RPM were wet processed, i.e., scrubbing, washing, and screened into various fractions by the wet processing recycling facility. The grain size of the fractions used in the study is 4–16 mm as shown in Figure 1. RPM was blended by weight with REM, thus the notation 0 RPM–100 REM refers to 0% RPM and 100% REM, 25 RPM–75 REM refers to 25% RPM and 75% REM, 50 RPM–50 REM refers to 50% each, whereas 100 RPM–0 REM refers to 100% RPM and 0% REM.

The main geological composition of REM is feldspathic rock, granite, and gneiss, with an occasional presence of phyllites. REM shows predominant minerals of quartz, and feldspar group of minerals, i.e., plagioclase and microcline/orthoclase. In addition, a low amount of mica minerals of muscovite and biotite group and almost insignificant amount of clinochlore were found. The minerals present in RPM are feldspar (i.e., typical traces of anorthite and microcline/orthoclase), quartz, chlorite (mainly clinochlore), and mica group (i.e., biotite, muscovite).

**Figure 2:**
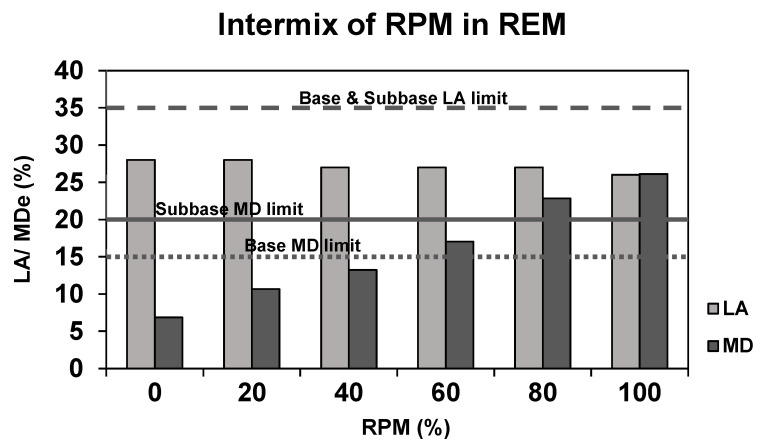
LA and MD performance of REM and mixed with RPM, adapted from [14].

The physical and mechanical performance of REM and RPM is shown in Table 1 and Figure 2, respectively. The Flakiness Index (FI) of the two materials was essentially different, as expected, due to the layered shape of phyllite rocks. However, considering RA produced from CDW, FI is reported in the region 8–30% [36]. The water absorption of REM and RPM did not vary significantly, and both materials had the same particle density values. Water absorption and particle density values obtained for RA were reported to be 4–6% and 2.3–2.4 kg/cm^3^, respectively [36].

The LA and MD are critical mechanical properties to assess the strength of aggregates applied in road pavement. The LA determines the resistance to fragmentation, whereas MD evaluates wear resistance. The LA values for REM and RPM were 28% and 26%, respectively, whereas the MD was reported to be 6% and 26% [14]. The MD results varied significantly between REM and RPM. Compared to the performance of materials for base and subbase application defined by the Norwegian Public Roads Administration [37], the LA values met the base course and subbase limit requirement at ≤35%. Regarding the MD, only RPM exceeded the limit criteria of ≤15% for base and ≤20% for subbase. Hence, to comply with MD, the maximum content of RPM was found to be around 40%, see Figure 2.

**Figure 3:**
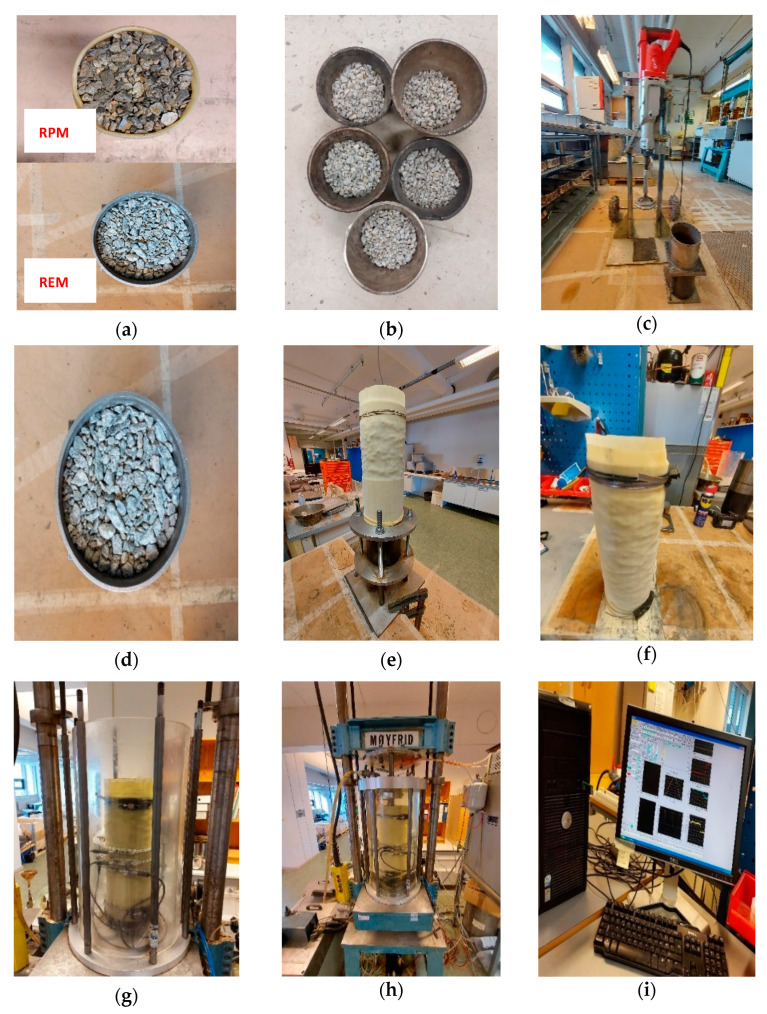
Repeated Load Triaxial Test set up. (**a**) Sample of REM and RPM; (**b**) Sample distribution into five different parts following specific gradation; (**c**) Vibrator hammer for sample compaction; (**d**) Fully compacted specimen in the steel mold; (**e**) Ejected specimen covered with first latex membrane; (**f**) Mounting of second latex membrane, plastic rings, and hose clamps; (**g**) Specimen placed in RLTT chamber with mounted two vertical and three horizontal linear variable differential transducers (LVDTs); (**h**) RLTT chamber filled with water and ready to begin test; (**i**) Computer setup for data collection.

Regarding the sample preparation for RLTT, a bulk mass of 8.500 g was measured in different quantities for pure REM and RPM based on the gradation specified in Figure 1. In addition, 25% and 50% RPM blended batches were prepared. Fractions of REM and REM are shown in Figure 3a. Each bulk sample consisted of fractions obtained from the predefined gradation groups of 16–11.2 mm, 11.2–8 mm, 8–4 mm, to represent an effective distribution of particles. The distribution followed five individual groupings, (see Figure 3b for distribution example of REM), and each part was carefully placed into the mold and compacted. Milwaukee 2″SDS Max rotary hammer (hammer weight 12 kg, work per blow 27 Nm, tamping time 25 s) was used to compact each layer in the steel mold (see Figure 3c). The compacted specimen ready to be ejected from the mold is shown in Figure 3d. This operation requires special attention to avoid losing any particle, as shown in Figure 3e. Finally, the specimen is covered by two latex membrane and supported by two endplates, four plastic rings and two hose clamps to avoid penetration of water, see Figure 3f. Two Linear Variable Differential Transducers (LVDTs), are mounted to measure axial deformation, and three LVDTs measure the radial deformation, see Figure 3g. The complete test setup with the chamber filled with water is shown in Figure 3h, and Figure 3i shows the computer for data collection.

**Figure 4:**
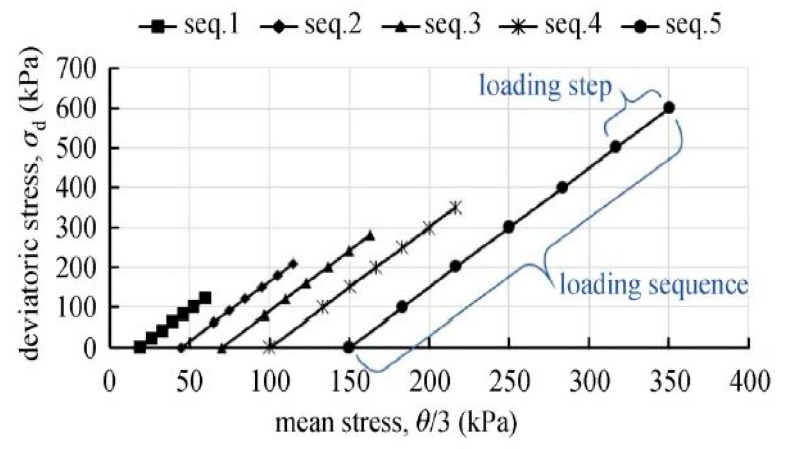
Loading sequence and load steps in a Multi-Stage Low-Stress level State (MS LSL), adapted from [39].

The RLTT consists of a Multi-Stage Low-Stress Level (MS LSL) loading procedure on the specimen, according to the European Standard EN 13286-7 [38]. The process involves applying two stress paths, i.e., pressurized water acting in all directions and the hydraulic jack acting vertically. Thus, the pressurized water exerts a constant confining stress (triaxial stress, *σ_t_*) at different stress levels. The hydraulic jack produces vertical dynamic stress (deviatoric stress, $\sigma_{d}$) following a sinusoidal pattern and increases stepwise at each sequence of $\sigma_{t}$. A minimum pressure value of 5 kPa produces contact between the hydraulic jack and the specimen. The loading sequence follows five incremental stages of $\sigma_{t}$ at 20, 45, 70, 100, and 150 kPa, with six varying load steps ($\sigma_{d}$). At each load sequence, about 10,000 load cycles of 10 Hz frequency result in a single load step (see Figure 4). In addition, Figure 4 indicates the loading sequence and load step in connection to the bulk stress θ (θ = $\sigma_{1}$+ $\sigma_{2}$+$\sigma_{3}$ = $\sigma_{d}$ + 3$\sigma_{t}$, where $\sigma_{1}$, $\sigma_{2}$, and $\sigma_{3}$ are principal stresses-both deviatoric and triaxial stress). When the permanent axial deformation reaches 0.5%, the deviatoric stress automatically stops, and the operator is now able set the next load sequence.

## 3. Results and Discussion

The following section discusses the resilient modulus and permanent deformation results obtained from the RLTT tests.

### Resilient Modulus (M_R_) of REM and RPM

The resilient modulus *M_R_* is the stiffness of materials and is given as the ratio between the change in deviatoric stress ($\Delta \sigma_{d}$) and the change in resilient or recoverable strain $\left(\Delta \varepsilon_{r}\right)$,

(1) $$M_{R}=\frac{\Delta \sigma_{d}}{\Delta \varepsilon_{r}}$$

**Figure 5:**
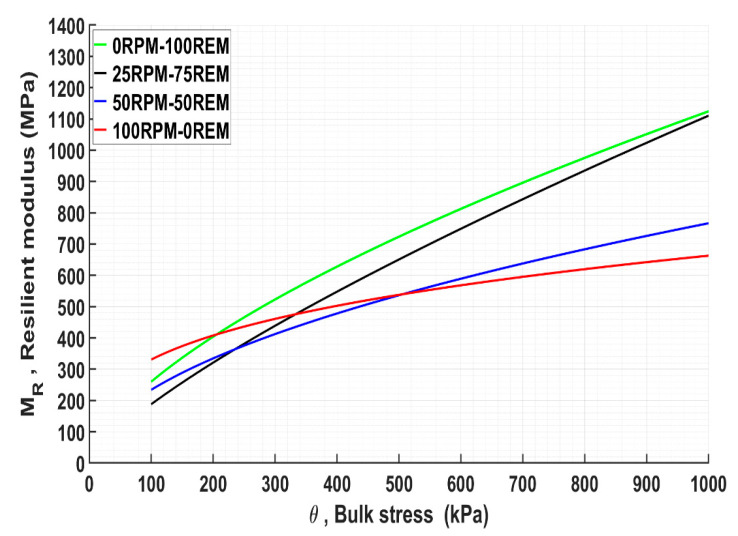
Resilient modulus of pure REM and RPM and blended mixtures by Hicks and Monismith model.

Hence, *M_R_* is related to the stress state and it is an important parameter for pavement design. Several nonlinear models describe the relationship between *M_R_* and θ. The most common formulation is the Hicks and Monismith’s model [40], expressed in its dimensionless form:

(2) $$M_{R}=k_{1}\;\sigma_{a}{(\frac{\theta }{\sigma_{a}})}^{k_{2}}$$

where *k*_1_ and *k*_2_ are regression model parameters generated from the test results, $\sigma_{a}$ is a reference pressure taken to be the same as atmospheric pressure (100 kPa), and θ is the bulk stress. The model expresses the performance of the materials in a two-dimensional state. In the present study, the *M_R_* was calculated by this approach, and the result is presented in Figure 5. Uzan’s model, which describes *M_R_* as a function of θ and σ presents a three-dimensional plot [41] and, therefore, it is another valuable approach to compare the behavior of materials. The three parameters indicated in Uzan’s model are (*M_R_*, θ, $\sigma_{d}$) and the model is expressed by:

(3) $$M_{R}=k_{1}\;\sigma_{a}{(\frac{\theta }{\sigma_{a}})}^{k_{2}}{(\frac{\sigma_{d}}{\sigma_{a}})}^{k_{3}}$$

where *k*_1_, *k*_2_, *k*_3_ are regression parameters.

As already mentioned above, moisture content represent one of the sensitive factors that affect the stiffness and PD behavior of UGM. Some studies have shown that moisture content significantly influenced the performance of RA at high-stress levels [28,30]. In addition, it is demonstrated regarding phyllite materials that they are sensitive to moisture variations, and as a result, less cohesion and rapid development of plasticity may occur [42]. Given this, our test was performed on dry batches of REM and RPM.

The stiffness performance of unblended and blended mixtures of REM and RPM at different θ is shown in Figure 5. Slightly high stiffness of 330 MPa was observed at the baseline 100 RPM compared with 260 MPa for 100 REM, at θ of 100 kPa. Foliated rocks of metamorphic origin composed of fine-grained felsic, and a significant amount of mica showed less stiffness of approximately 200 MPa at the same θ of 100 kPa [17]. Nevertheless, increased stiffness of 100 REM occurs at θ starting from 200 kPa, and the performance assumed better cohesion and frictional properties as the stiffness behaved almost linearly. This observation may be due to a considerable increase of *M_R_* properties as the confinement pressure and consolidation increased. A possible explanation for slightly high *M_R_* for 100 RPM at low bulk stress may be due to the slow packing density and particle re-orientation at the initial stage of the compaction process. In addition, the high surface area between the particles may have contributed to the initial performance since RPM is characterized by high FI. The stiffness response of both materials in a pure state indicates the vital role of material properties on performance.

For the blended mixtures, the stiffness decreased with an increased RPM content. 25 RPM–75 REM blends showed a performance pattern similar to 100 REM, whereas 50 RPM–50 REM performed similarly to 100 RPM. At a high-stress state, blended and unblended mixture 25 RPM–75 REM and 100 REM respectively provide higher stiffness performance. A similar conclusion was reached regarding the quantity of weak material in a mix for sufficient stiffness performance in the study [28]. In their study, the effect of CB in mixtures 25 CB–75 RCA and 25 CB–75 CR was consistent, except that the cemented part of RCA could potentially lead to significant permanent strain due to residual cement action. However, the authors mentioned that it is unlikely that such changes would significantly affect pavement performance over time. The LA values obtained in that study were 21%, 28%, and 36% for CR, RCA, and CB, respectively [28].

In other studies, effective stiffness response of RAP and RCA blends was achieved at 15 RAP–85 RCA combinations [29], and the LA of both materials in the pure state was reported as 42% and 28%, respectively. The LA for the mixture was 38% [29]. A comparative study of deformation and stiffness behavior of two RCA and virgin quartzite rocks showed that the stiffness of RCA increased within the region of 490–1020 MPa, whereas quartzite performed within the region 480–685 MPa [43]. According to the authors, both materials’ performance complied with the Australian requirement of 300 MPa, and the reported LA was 39% and 37% for both RCA and 25% for quartzite [43]. As found in the present study, the stiffness behavior of REM and RPM follows a similar pattern of mafic igneous rocks and metamorphic rocks classified as strong and weak rocks, respectively [17]. The authors mentioned that metamorphic rocks consisted of fine-grained felsic and micaceous texture, and the LA values reported were 17% and 30% for mafic igneous and metamorphic rocks, respectively. In the Netherlands, the stiffness values for natural aggregates, RMA, MRA and RCA are reported to be 100–400 MPa, 150–250 MPa, 400–600 MPa, and 600–800 MPa, respectively [44].

Given the baseline performance reported in the studies above, it is sufficient to mention that both REM and RPM showed optimal stiffness responses despite the significant performance variation between the two, which is certainly due to the inherent properties of the materials.

It is crucial to emphasize the effect of shape particle and FI regarding the performance of RPM. Although this was not experimentally demonstrated in this study, several researchers have investigated the effect of shape on the stiffness and deformation properties of unbound materials. As already shown in Table 1, a significant variation of FI performance was found between REM and RPM. Both materials meet the FI requirement reported as ≤35% by the Norwegian Public Roads Administration [37]. A study of gneiss rocks with different textural and shape properties (flaky, flaky rounded, cubic, and cubic rounded) was performed [45], and the results showed that for the same θ of 200 kPa, cubic and cubic rounded particles had the highest stiffness performance in the region of 300–400 MPa, whereas that of flaky particles was ˂300 MPa. It was observed in the same study that as the θ increased to 1000 kPa, a marginal drop of stiffness in the cubic particles was observed; nevertheless, the study concluded that cubic particles showed highest stiffness response. Additionally, for recycled aggregates, some authors have established strong relationships between FI and stiffness and deformation properties [46,47]. Hence, it is necessary to study the effect of other physical properties and, in this case, FI to understand the behavior altogether.

Uzan’s model presents a three-dimensional view plot of the relationship between *M_R_* θ and $\sigma_{d}$ as discussed above. The model was applied to the RLTT data, and the regression parameters used in both Hicks and Monismith’s and Uzan’s models are shown in Table 2.

**Figure 6:**
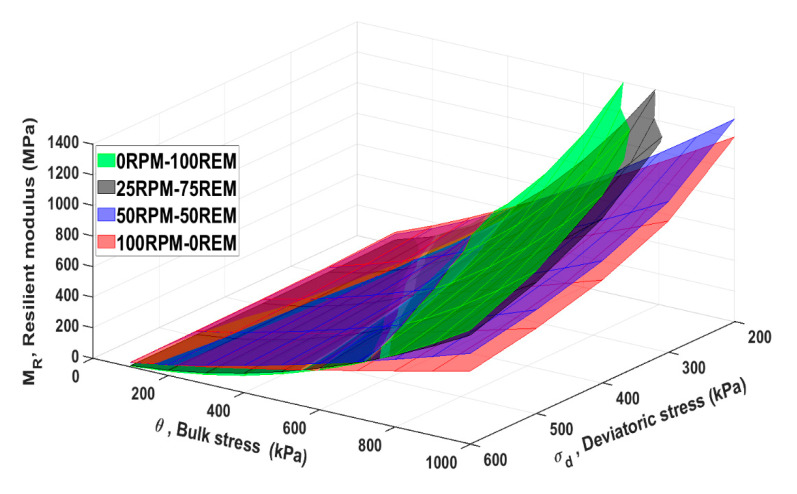
Resilient modulus of pure REM and RPM and blended mixtures by Uzan’s model.

Regarding Uzan’s model, the results showed similar performance of *M_R_* for the materials in pure and mixed conditions, see Figure 6.

#### Permanent Deformation Behavior of REM and RPM

**Figure 7:**
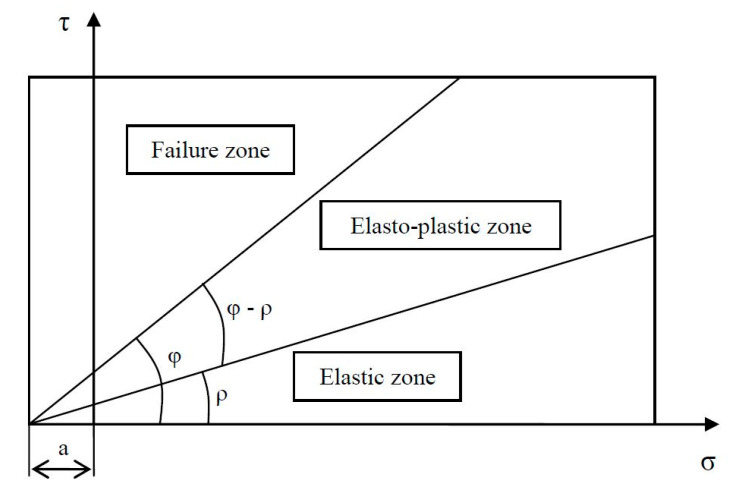
Material behavior classified according to mobilized shear strength *ρ*(°) and maximum shear to failure *φ*(°) in three ranges. The Coulomb approach applied to cyclic load triaxial testing is demonstrated in a τ-σ plot, adapted from [45].

In this study, the Coulomb approach was adopted to investigate the PD of the materials. The Coulomb approach, which derives from the shakedown approach characterizes the mobilized friction angle *ρ*(°) and incremental friction angle *φ*(°), which describes the degree of mobilized and maximum shear strength, respectively [48] as shown in Figure 7. These two angles illustrate the behavior of materials according to three ranges, i.e., elastic, elasto-plastic, and failure, see Figure 7. Table 3 shows the permanent strain rate for each performance range.

The accumulation of PD is classified into the three performance ranges according to the aforementioned angles in the Coulomb model and is represented by best-fit lines. Each load step is defined by the average strain rate *ἐ* developed within 5.000 to 10.000 load cycles [48], which is described as a measure of the speed to PD [45]. Regarding the elastic limit and failure limit, they are respectively defined by the following equations.

(4) $$\sigma_{d}=\frac{2\sin\rho \;\left(\sigma_{3}+a\right)}{1-\sin\rho }$$

(5) $$\sigma_{d}=\frac{2\sin\varphi \;\left(\sigma_{3}+a\right)}{1-\sin\varphi }$$

where a is the apparent attraction considered equal to 20 kPa [45].

**Figure 8:**
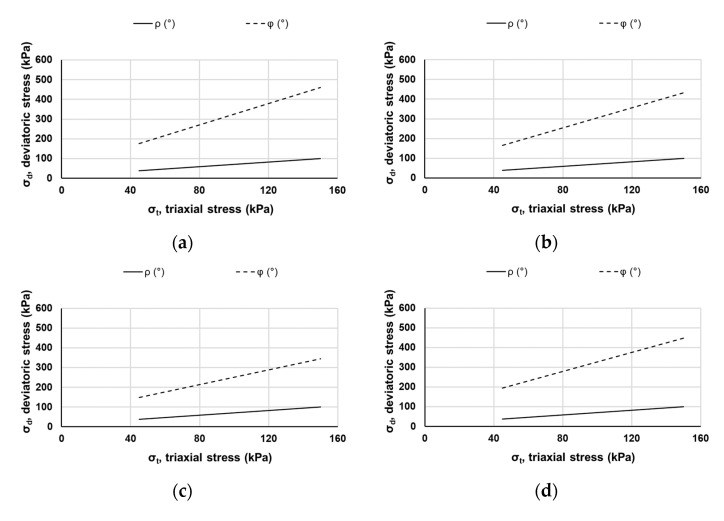
Degree of mobilized shear strength *ρ*(°) and maximum shear strength *φ*(°) of (**a**) 0% RPM–100% REM; (**b**) 25% RPM–75% REM; (**c**) 50% RPM–50% REM; and (**d**) 100% RPM–0% REM.

**Figure 9:**
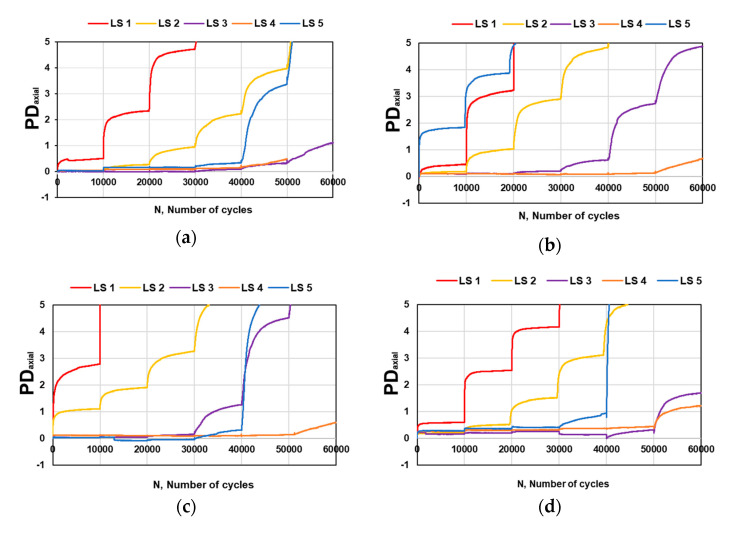
Permanent deformation (PD) of REM and RPM as function of the load cycles (N) for (**a**) 0% RPM–100% REM; (**b**) 25% RPM–75% REM; (**c**) 50% RPM–50% REM, and (**d**) 100% RPM–0% REM.

The mobilized friction angle *ρ*(°) and incremental friction angle *φ*(°) of REM and RPM in unblended and blended mixtures resulting from the loading sequence are shown in Figure 8, and Table 4 illustrates the limit values obtained for *ρ*(°) and *φ*(°), respectively. To present a typical raw data trend, Figure 9 shows the pattern of accumulated axial permanent deformation (PDaxial) for each of the five loading sequences (LS) in each tested mix percentage. There is no variation in the degree of mobilized angle for the samples as the values are the same. Regarding the incremental friction angle *φ*(°), 50% RPM–50% REM batch had the lowest value. Overall, the samples tested in this study offered a similar response when the resistance to PD was determined. Unlike stiffness, blended mixtures assessed for PD behavior did not show sensitivity to increased RPM content. Comparing the performance to crushed rocks in the studies [17,39,49], the values of mobilized friction angle *ρ*(°) obtained in this study is small; nevertheless, the incremental friction angle *φ*(°) is similar to the results obtained in the studies mentioned above. Such changes could be the differences in material properties, as emphasized by [49] that differences in the grading envelope reflected the discrepancies in the PD behavior. A basic friction angle for unweathered rock surfaces falls between 25° and 35° [50]. Regarding RA, it was found that the friction angle was small in the blends of 25 CB–75 RCA and 25 CB–75 CR [28].

The PD of the materials gave a true reflection of the performance concerning degradation behavior in the LA tests. There is a strong relationship between PD, fine particles, and high fouling index [51]. In other words, rocks susceptible to fragmentation or crushing may develop a significant number of fine particles over time, resulting in excessive PD. Similarly, a conclusion was reached that angularity and surface texture positively influenced the elastic and plastic shakedown thresholds for cubic aggregates [45]. Based on these findings, it is possible to infer that the PD performance of both REM and RPM are satisfactory. Therefore, the use of REM with some amount of RPM should not be considered detrimental to overall performance.

## 4. Conclusions

This work investigated the resilient modulus (*M_R_*) and permanent deformation (PD) behavior of Recycled Excavation Materials (REM) blended with Recycled Phyllite Materials (RPM) by Repeated Load Triaxial Test (RLTT). RPM was systematically incorporated into REM at 0%, 25%, 50%, and 100% to document the maximum substitution ratio without compromising the performance. Hicks and Monismith’s model and Uzan’s model assessed the stiffness, while the Coulomb model was used to evaluatethe PD.

The investigation findings showed that the stiffness behavior of REM and RPM in a pure state varied considerably, with 0% RPM–100% REM exhibiting high stiffness strength. Regarding blended mixtures, 25% RPM–75% REM achieved almost the same strength characteristics as REM in a pure state, whereas 50% RPM–50% REM performed similarly to RPM. The behavior of the materials in this regard showed sensitivity to the increased content of RPM.

The PD response of both materials did not show significant differences as the degree of mobilized friction angle *ρ*(°) for the samples was the same. Regarding the incremental friction angle *φ*(°), 50% RPM–50% REM had the lowest value; however, no sensitivity to increased RPM content in the PD was shown. The study’s findings may be helpful to end-users regarding the maximum quantity of RPM in REM considering the application of REM in unbound construction.

Given the influence of physical properties, as demonstrated in other studies on the stiffness and deformation characteristics of unbound materials, the authors recommend that future research should focus on the effect of material properties such as the shape and flakiness index of unblended and blended REM and RPM mixtures.

## Figures and Tables

**Table 1:** Physical properties of REM and RPM given as the arithmetic mean ± 1 standard deviation, n = 3.

Sample Name	Flakiness Index (%) EN 933-3	Water Absorption (%) EN 1097-6	Particle Density (g/mL) EN 1097-6
REM	12.6 ± 2.5	0.3 ± 0.2	2.5 ± 0.1
RPM	31.1 ± 1.3	0.6 ± 0.1	2.5 ± 0.1

**Table 2:** Regression parameters of Hicks and Monismith’s and Uzan’s models.

Sample	Hicks and Monismith		Uzan		
	*k*_1_	*k*_2_	*k*_1_	*k*_2_	*k*_3_
0 RPM–100 REM	2363	0.59	779	1.66	−0.94
25 RPM–75 REM	1881	0.77	575	1.88	−1.01
50 RPM–50 REM	2597	0.44	1026	1.30	−0.76
100 RPM–0 REM	4122	0.29	1306	1.46	−1.11

**Table 3:** Classification of the deformation behaviour according to Coulomb model.

Permanent Strain Rate (*ἐ*)	Performance Range
*ἐ* ˂ 2.5 × 10^−8^	elastic range
2.5 × 10 ^−8^ ˂ *ἐ* ˂ 1.0 × 10^−7^	elasto-plastic range
*ἐ* ˃ 1.0 × 10^−7^	failure range

**Table 4:** Mobilized angle of friction *ρ*(°) and incremental angle to failure *φ*(°) for REM and RPM in pure and blended state.

Sample	Limit Angles	
	*ρ*(°)	*φ*(°)
0 RPM–100 REM	30.4	69.7
25 RPM–75 REM	30.4	68.5
50 RPM–50 REM	30.4	66.4
100 RPM–0 REM	30.4	71.4

## Data Availability

The data presented in this paper is available upon request from the corresponding author.

## Abbreviations

CB	Crushed Brick
CDW	Construction and Demolition Waste
CDRA	Construction and Demolition Waste Recycled Aggregates
CR	Crushed Rock
FI	Flakiness Index
LA	Los Angeles Test
LSL	Low-Stress Level
LVDT	Linear Variable Differential Transducers
MD	Micro-Deval Test
MS	Multi-Stage
MRA	Mixed Recycled Aggregates
MR	Resilient Modulus
PD	Permanent Deformation
PDaxial	Axial Permanent Deformation
RA	Recycled Aggregates
RAP	Recycled Asphalt Pavement
RCA	Recycled Concrete Aggregates
REM	Recycled Excavation Materials
RLTT	Repeated Load Triaxial Test
RMA	Recycled Masonry Aggregates
RPM	Recycled Phyllite Material
UGM	Unbound Granular Material
